# From screening to postpartum follow-up – the determinants and barriers for gestational diabetes mellitus (GDM) services, a systematic review

**DOI:** 10.1186/1471-2393-14-41

**Published:** 2014-01-22

**Authors:** Karoline Kragelund Nielsen, Anil Kapur, Peter Damm, Maximilian de Courten, Ib Christian Bygbjerg

**Affiliations:** 1 Department of International Health, Immunology and Microbiology, University of Copenhagen, Oester Farimagsgade 5, Building 9, Copenhagen DK-1014, K, Denmark; 2 World Diabetes Foundation, Brogaardsvej 70, Gentofte DK–2820, Denmark; 3 Center for Pregnant Women with Diabetes, Department of Obstetrics, Rigshospitalet, University of Copenhagen, Blegdamsvej 9, Copenhagen DK-2100, Denmark

**Keywords:** Gestational diabetes mellitus, Health services, Barriers, Determinants, Lifestyle, Screening

## Abstract

**Background:**

Gestational diabetes mellitus (GDM) – a transitory form of diabetes first recognised during pregnancy complicates between < 1% and 28% of all pregnancies. GDM has important short and long-term health consequences for both the mother and her offspring. To prevent adverse pregnancy outcomes and to prevent or delay future onset of type 2 diabetes in mother and offspring, timely detection, optimum treatment, and preventive postpartum care and follow-up is necessary. However the area remains grossly under-prioritised.

**Methods:**

To investigate determinants and barriers to GDM care from initial screening and diagnosis to prenatal treatment and postpartum follow-up, a PubMed database search to identify quantitative and qualitative studies on the subject was done in September 2012. Fifty-eight relevant studies were reviewed.

**Results:**

Adherence to prevailing GDM screening guidelines and compliance to screening tests seems sub-optimal at best and arbitrary at worst, with no clear or consistent correlation to health care provider, health system or client characteristics. Studies indicate that most women express commitment and motivation for behaviour change to protect the health of their unborn baby, but compliance to recommended treatment and advice is fraught with challenges, and precious little is known about health system or societal factors that hinder compliance and what can be done to improve it. A number of barriers related to health care provider/system and client characteristics have been identified by qualitative studies. Immediately following a GDM pregnancy many women, when properly informed, desire and intend to maintain healthy lifestyles to prevent future diabetes, but find the effort challenging. Adherence to recommended postpartum screening and continued lifestyle modifications seems even lower. Here too, health care provider, health system and client related determinants and barriers were identified. Studies reveal that sense of self-efficacy and social support are key determinants.

**Conclusions:**

The paper identifies and discusses determinants and barriers for GDM care, fully recognising that these are highly dependent on the context.

## Background

Affecting between <1% and 28% of all pregnancies gestational diabetes mellitus (GDM) is a fairly common medical condition associated with pregnancy in many settings [1]. As the epidemic of diabetes and pre-diabetes in the general population grows with declining age of onset and increasing age of conception and child bearing, the rates of GDM will continue to rise as already seen in some studies [2-4]. GDM was earlier defined as “hyperglycaemia first recognized during pregnancy” and has more recently (2012) been described by the American Diabetes Association (ADA) as diabetes diagnosed during pregnancy that is not clearly overt diabetes [5]. GDM has health consequences for both the mother and her offspring not only in the short term but also in the long term. It is well established that women with GDM are at increased risk of adverse pregnancy outcomes [6,7], as well as, several fold higher risk of developing type 2 diabetes in the future compared to women without GDM [8,9]. Studies show that achieving glycaemic control with lifestyle modifications and/or pharmaceutical intervention during pregnancy prevents or considerably reduces the risk of adverse pregnancy outcomes [10,11]. Clinical trials provide evidence that lifestyle modifications as well as pharmacological interventions can prevent progression to type 2 diabetes in women with a history of GDM and these interventions are as effective as in people with pre-diabetes [12-14]. Effective intervention requires universal antenatal screening for GDM, optimal treatment and adherence, and rigorous postpartum follow-up and preventive care. Nielsen et al. recently pointed out the urgent need for universally applicable simple screening and diagnostic procedures criteria for GDM [15]. In this paper we attempt to identify determinants and barriers to implementing effective and integrated public health initiatives to address screening, diagnosis, treatment and postpartum care for GDM based on a review of published studies on the subject.

## Methods

We reviewed the literature to assess existing evidence on determinants and barriers for GDM services in high-, middle- and low-income countries. GDM services were understood as 1) screening and diagnosis 2) treatment during pregnancy 3) postpartum diabetes testing and 4) continuation of postpartum lifestyle modification.

### Search strategy

PubMed database was searched in September 2012 using the terms “GDM/gestational diabetes mellitus/‘diabetes’ and ‘pregnancy’” in combination with one of the terms “barrier/barriers/challenge/challenges/determinant/determinants/utilization/use/access/lifestyle change/self-efficacy/social support”. The Mesh terms “gestational diabetes mellitus” and “access to health care” were also used.

### Study selection, eligibility criteria and data abstraction

Studies that only examined pre-gestational diabetes whether type 1 or type 2 diabetes were excluded as were studies where specific information on women with GDM was not available. The list of citations was scanned and qualitative and quantitative research studies with relevant titles examining determinants and/or barriers to GDM services and related aspects, e.g. compliance to treatment, were included. The abstracts of these studies were read and those with an abstract and subsequent full-text reading indicating relevance to the purpose of this review were selected. Finally, the reference lists of the selected publications were manually searched for additional relevant articles.

## Results

### Search outcome

**Figure 1:**
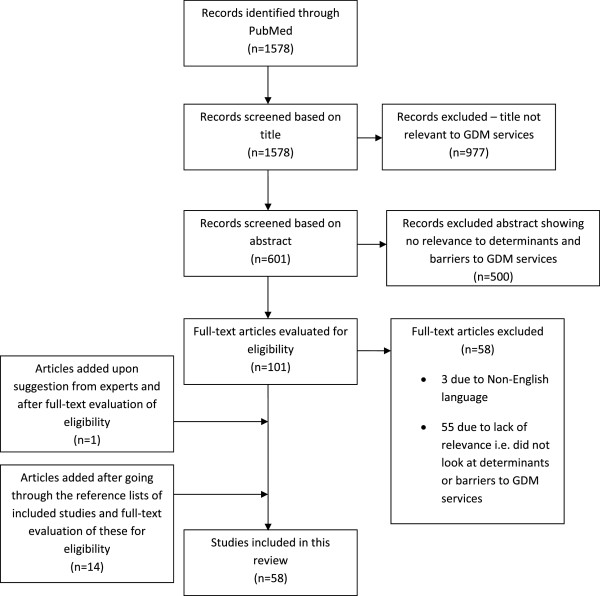
Flow chart of study search and selection.

A total of 1578 unique citations were identified; 977 were excluded based on the title and another 500 were excluded after reading the abstract. Of the remaining 101, three papers were non English language (1 from Poland, Spain and Mexico, respectively) and excluded. The remaining 98 articles were read for relevance for this review and a further 55 were excluded because of lack of relevance i.e. studies did not look at determinants or barriers to GDM services. One article was added following suggestion from experts and 14 others were added after going through the reference lists of included articles. Thus, a total of 58 papers were included in this review (see Figure 1).

The studies included are listed in Tables 1, 2 and 3. The majority are from high-income countries: USA (N = 28), Canada (N = 8), Australia (N = 10), New Zealand (=1) and European countries (N =7) and only four from low- and middle-income countries.

**Table 1:** **Overview of articles focusing on screening** (**n** = **12**) (**alphabetic order**)

**Article**	**Country**	**Study design**	**Study population**	**Focus**
Blatt et al. (2011) [23]	USA	Review of data from Quest Diagnostics	924,873 pregnant women	Characteristics of pregnant women
Cullinan et al. (2012a) [24]	Ireland	Hospital clinical data	9,842 pregnant women	Characteristics of the pregnant women
Cullinan et al. (2012b) [25]	Ireland	Hospital clinical data	4,414 pregnant women	Characteristics of the pregnant women
Gazmararian et al. (1996) [19]	USA	Review of prenatal care records	2,184 pregnant women	Characteristics of health care providers
				Characteristics of the pregnant women
Landon et al. (1990) [16]	USA	Cross-sectional mail survey	471 obstetricians and maternal-fetal specialists	Characteristics of health care providers
Marrero et al. (1992) [17]	USA	Mail survey	668 family practice physicians and obstetricians/gynaecologists	Characteristics of health care providers
Moses et al. (2003) [21]	Australia	Review of medical records	1,648 pregnant women	Characteristics of health care setting
Nielsen et al. (2012) [15]	Various developing countries	Qualitative. Interviews and questionnaire.	10 GDM project implementers	Barriers mentioned by project implementers
Pedula et al. (2009) [26]	USA	Review of medical records	21,758 pregnancies	Characteristics of the pregnant women
Ruengkhachorn (2006) [20]	Thailand	Review of antenatal care records	159 pregnant women	Characteristics of health care setting
				Characteristics of the pregnant women
Sievenpiper et al. (2012) [18]	Canada	Audit	1,026 pregnant women	Characteristics of health care providers
				Characteristics of the pregnant women
Yapa & Simmons (2000) [22]	New Zealand	Review of hospital medical records	4,885 pregnant women	Characteristics of the pregnant woman

**Table 2:** **Overview of articles focusing on treatment** (**n** = **15**) (**alphabetic order**)

**Article**	**Country**	**Study design**	**Study population**	**Focus**
Bandyopadhyay et al. (2011) [29]	Australia	Qualitative. Semi-structured interviews.	17 women with GDM	Experiences of living with GDM
				Barriers mentioned by women with GDM
Collier et al. (2011) [39]	USA	Qualitative. Focus group discussions.	4 focus group discussion with women with GDM	Barriers mentioned by women with GDM
Doran & Davis (2010) [30]	Tonga	Qualitative. Semi-structured interviews	11 women with GDM	Experiences of living with GDM
				Barriers mentioned by health care providers
				Barriers mentioned by women with GDM
Evans & O’Brien (2005) [31]	Canada	Qualitative. Interviews	12 women with GDM	Experiences of living with GDM
				Barriers mentioned by women with GDM
Hirst et al. (2012) [38]	Vietnam	Qualitative. Focus group discussions	4 focus group discussions with women with GDM	Experiences of living with GDM
				Barriers mentioned by women with GDM
Hjelm et al. (2007) [36]	Sweden	Qualitative. Semi-structured interviews	27 women with GDM	Experiences of living with GDM
				Barriers mentioned by women with GDM
Hjelm et al. (2008) [37]	Sweden	Qualitative. Semi-structured interviews	23 women with GDM	Experiences of living with GDM
				Barriers mentioned by women with GDM
Landon et al. (1990) [16]	USA	Cross-sectional mail survey	471 obstetricians and maternal-fetal specialists	Characteristics of health care providers
Lawson & Rajaram (1994) [32]	USA	Qualitative. Interviews	17 women with GDM	Experiences of living with GDM
				Barriers mentioned by women with GDM
Marrero et al. (1992) [17]	USA	Mail survey	668 family practice physicians and obstetricians/gynaecologists	Characteristics of health care providers
Mersereau et al. (2011) [40]	USA	Qualitative. Focus group discussions	6 focus groups with health care providers	Barriers mentioned by health care providers
Neufeld (2011) [33]	Canada	Qualitative. Semi-structured interviews	29 women with GDM	Experiences of living with GDM
				Barriers mentioned by women with GDM
Persily (1996) [35]	USA	Convenience sample of women with GDM followed from diagnosis through delivery	29 women with GDM	Experiences of living with GDM
Persson et al. (2010) [34]	Sweden	Qualitative. Interviews	10 women with GDM	Experiences of living with GDM
				Barriers mentioned by women with GDM
Ruggiero et al. (1990) [28]	USA	Cross-sectional survey	98 women with GDM	Role of psychosocial factors

**Table 3:** **Overview of articles focusing on postpartum follow**-**up** (**n** = **36**) (**alphabetic order**)

**Article**	**Country**	**Study design**	**Study population**	**Focus**
Almario et al. (2008) [41]	USA	Review of data from Laboratory Corporation of America and Quest Diagnostics	90 women with GDM	Characteristics of health care setting
				Characteristics of services/treatment received
				Characteristics of women with GDM
Baker et al. (2009) [48]	USA	Mail survey	399 health care providers	Characteristics of health care providers
				Barriers mentioned by health care providers
Bandyopadhyay et al. (2011) [29]	Australia	Qualitative. Semi-structured interviews.	17 women with GDM	Experiences of living with GDM
				Barriers mentioned by women with GDM
Beischer et al. (1997) [47]	Australia	Cohort study using data from GDM follow-up programme	2,939 women with GDM	Characteristics of health care setting
				Characteristics of services/treatment received
				Characteristics of women with GDM
Bennett et al. (2011) [61]	USA	Qualitative. Semi-structured interviews.	22 women with GDM	Barriers mentioned by women with GDM
Blatt et al. (2011) [23]	USA	Review of data from Quest Diagnostics	40,955 women with GDM	Characteristics of women with GDM
Clark et al. (2009) [55]	Canada	Randomized controlled trial	223 women with GDM	Characteristics of services/treatment received
Dietz et al. (2008) [46]	USA	Kaiser Permanente Northwest data	461 women with GDM for first part of the study and 356 for second part.	Characteristics of health care setting
				Characteristics of services/treatment received
				Characteristics of women with GDM
Ferrara et al. (2009) [58]	USA	Review of medical records	14,448 women with GDM	Characteristics of women with GDM
				Characteristics of services/treatment received
Gabbe et al. (2004) [49]	USA	Mail survey	569 obstetricians/gynaecologists	Characteristics of health care providers
Graco et al. (2009) [70]	Australia	Qualitative. Interviews	10 women with GDM	Barriers mentioned by women with GDM
Hoedjes et al. (2012) [62]	The Netherlands	Qualitative. Focus group discussions	5 women with GDM	Barriers mentioned by women with GDM
Hunt & Conway (2008) [59]	USA	Prospective cohort study	707 women with GDM	Characteristics of women with GDM
				Characteristics of services/treatment received
Kaufmann et al. (1999) [53]	USA	Cross-sectional survey	66 women with GDM	Characteristics of health care providers
				Characteristics of women with GDM
Keely et al. (2010) [50]	Canada	Fax and telephone survey	173 primary care providers and 140 women with GDM	Barriers mentioned by health care providers
				Barriers mentioned by women with GDM
Kim et al. (2008) [67]	USA	Cross-sectional survey	228 women with GDM	Role of psychosocial factors
Kim et al. (2007) [57]	USA	Cross-sectional survey	217 women with GDM	Experience of risk of future diabetes
Kim et al. (2007a) [65]	USA	Cross-sectional survey	228 women with GDM	Characteristics of services/treatment received
Kim et al. (2006) [42]	USA	Review of hospital medical records	533 women with GDM	Characteristics of women with GDM
				Characteristics of health care providers
				Characteristics of services/treatment received
Koh et al. (2010) [68]	Australia	Cross-sectional telephone survey	331 women with GDM	Characteristics of women with GDM
				Role of psychosocial factors
Kwong et al. (2009) [60]	Canada	Retrospective cohort study	909 women with GDM	Characteristics of women with GDM
				Characteristics of services/treatment received
Lawrence et al. (2010) [43]	USA	Kaiser Permanente Southern California Medical Care Program data	11,825 women with GDM	Characteristics of services/treatment received
				Characteristics of women with GDM
Morrison et al. (2009) [49]	Australia	Cross-sectional mail survey	1,372 women with GDM	Characteristics of health care providers
				Characteristics of women with GDM
				Characteristics of services/treatment received
Neufeld (2011) [33]	Canada	Qualitative. Semi-structured interviews	29 women with GDM	Experiences of living with GDM
				Barriers mentioned by women with GDM
Nicklas et al. (2011) [71]	USA	Qualitative. Interviews and focus group discussions	3 focus group discussions with women with GDM, and interviews with 15 women with GDM	Barriers mentioned by women with GDM
Razee et al. (2010) [72]	Australia	Qualitative. Semi-structured interviews.	57 women with GDM	Barriers mentioned by women with GDM
Russell et al. (2006) [54]	USA	Retrospective cohort study	344 women with GDM	Characteristics of health care setting
				Characteristics of women with GDM
Shah et al. (2011) [51]	Canada	Population-level health care database	47,691 women with GDM	Characteristics of health care providers
Shea et al. (2011) [56]	Canada	Hospital laboratory and provincial physician service claims databases	262 women with GDM	Characteristics of services/treatment received
				Characteristics of women with GDM
Smirnakis et al. (2005) [45]	USA	Review of medical records	197 women with GDM	Characteristics of health care providers
				Characteristics of women with GDM
Smith et al. (2005) [64]	Australia	Telephone survey	226 women with GDM	Barriers mentioned by women with GDM
				Role of psychosocial factors
Stage et al. (2004) [66]	Denmark	Mail survey	121 women with GDM	Experience of risk of future diabetes
Stuebe et al. (2010) [52]	USA	Survey	207 primary care providers and obstetrics & gynaecology care providers	Characteristics of health care providers
				Barriers mentioned by health care providers
Swan et al. (2007) [69]	Australia	Mail survey	53 women with GDM	Characteristics of women with GDM
Symons Downs & Ulbrecht (2006) [73]	USA	Mail survey	28 women with GDM	Barriers mentioned by women with GDM
Zehle et al. (2008) [63]	Australia	Telephone survey	226 women with GDM	Role of psychosocial factors
				Characteristics of women with GDM

### Determinants and barriers for GDM screening

Ensuring timely detection of GDM is a prerequisite for initiation of treatment and prevention of adverse outcomes due to GDM. Only a few relevant studies related to GDM screening were identified, and they mostly looked at determinants of compliance to screening recommendations.

### Characteristics of health care providers and health care setting

In the US over 20 years ago with various screening approaches available and no clear consensus on which one to follow, Marreo et al. and Landon et al. conducted two explorative studies on GDM screening and care practices [16,17]. Marrero et al. examined practice difference between family physicians and physicians specialising in obstetrics/gynaecology and found that the former were less likely to screen all pregnant women for GDM compared to latter (75% vs. 83%; P = 0.033) [17]. Landon et al. investigated differences in screening practices among members of the Society of Perinatal Obstetricians (SPO) and members of the American College of Obstetricians and Gynaecologists (ACOG) and found that a significantly higher proportion of the former practiced universal screening for GDM compared to the latter [16]. A more recent study by Sievenpiper et al. [18] looked at whether the category of health care provider ordering a 2-step test as recommended by the Canadian Diabetes Association influenced if the recommendation was followed appropriately i.e. whether an oral glucose tolerance test (OGTT) was completed and found no association. Gazmararian et al. [19] assessed whether the gender of the obstetrician and years since graduation influenced if the glucose challenge test was undertaken, and found that the likelihood increased if the obstetrician had graduated within the past 30 years, while gender had no significant effect. The authors acknowledged that glucose challenge test recommendations were not universally adhered to, but did not believe that time since graduation explained the differences in test executions.

A rather unusual finding was reported by Ruengkhachorn et al. from Thailand, where compliance to screening guideline recommending universal screening was 88% in women at risk of GDM attending a non-private hospital setting in Bangkok, but only half amongst women attending the antenatal care (ANC) clinic at a private hospital and none amongst those attending a private clinic. The authors hypothesised that the difference may be due to concerns of inconvenience, cost of test or negligence of the physicians themselves, and noted that a screening guideline had not been fully implemented at the private hospital [20]. Contrary to this, Moses et al. in their study from Australia where screening of all pregnant women is recommended found that women attending ANC at public hospitals were less likely to be screened for GDM compared to women attending ANC at general practitioners’ or obstetricians’ private practices [21].

### Client characteristics: ethnicity, body weight and age

#### Ethnicity

A study from New Zealand – a country with universal screening recommendation for GDM, found that only half of the women attending ANC were screened; being of non-European ethnicity significantly increased the likelihood; but still a large proportion of women with risk factors were not screened [22]. Ethnicity was also identified as a significant determinant for GDM screening by Blatt and colleagues in their study from the US, where non-white ethnicity increased the likelihood of being screened [23]. Two studies by Cullinan and colleagues have investigated determinants of GDM screening in Ireland where no single policy on GDM screening is implemented, but the local authorities in the region where the studies were conducted advocate a universal screening approach. The data in the studies are overlapping in the sense that one paper uses data from five health centres in West Ireland whereas the other paper only includes data from the largest of these five centres. Ethnicity was not found to be a statistically significant determinant in either of the papers [24,25]. Pedula and colleagues reported differences in the proportion screened among different ethnic groups in a hospital setting in Hawaii with a policy that GDM screening should be routinely done among pregnant women [26].

#### BMI and body weight

The American College of Obstetrics and Gynecologists recommends that all pregnant women without known diabetes should have their risk for GDM assessed by reviewing their history, clinical risk factors, including overweight/obesity, or by having laboratory screening tests done [27]. Yet, in their study from the US, Blatt et al. found that women weighing more than 275 pounds (125 kg) were 12% less likely to have a glucose tolerance test than the women in the reference group who weighed 100–124 pounds (45–56 kg) [23]. However, whether the women in the higher weight category had their clinical risk factors assessed and subsequent intervention initiated is not stated. Cullinan et al. found in both their studies that having a BMI over 30 kg/m^2^ significantly increased the likelihood of screening although this was not the case for overweight women with a BMI between 25 and 30 [24,25]. Likewise, Yapa & Simmons in their study from New Zealand also found that higher body weight significantly increased the likelihood of screening in an environment where universal screening is recommended [22].

#### Age

Several studies have investigated age as a determinant for screening. Despite the policy stating that GDM screening should be routinely done among pregnant women, Pedula et al. found that a higher proportion of younger women (≤ 25 years) rather than older women were screened [26]. Cullinan et al. and Blatt et al. found that older women were more likely to be screened than their younger counterparts [23-25]. In Cullinan et al.'s studies universal screening was advocated locally but not nationally, whereas in the study by Blatt et al. the recommendation is that women should have a glucose test or their clinical risk factors/patient history assessed. In both cases it could be hypothesised that age influences probability of screening, indicating an underlying tendency to rely on risk factor based screening. On the other hand, Yapa & Simmons did not find a significant association between age and screening for GDM in their setting where universal screening was recommended [22]. Similarly, Sievenpiper et al. found that maternal age did not affect the probability that the recommended 2-step approach was followed, as also the study by Gazmararian et al. who found no statistically significant association between maternal age and screening for GDM in an environment with no consensus on the screening approach [18,19].

#### Other factors

Gazmararian et al. also investigated whether marital status and trimester of entry to ANC influence the likelihood of a glucose challenge test being administered, but did not find a statistically significant association [19]. Cullinan and colleagues noted that greater distance to the screening site, residing in an urban, richer part of town and high parity decreased the likelihood of participating in screening in a setting where universal screening was advocated. In addition, a positive family history of diabetes did not significantly affect the decision to attend GDM screening [24,25]. Furthermore, Sievenpiper et al. found that a borderline result of the 50 g glucose challenge test (GCT) did not predict if an OGTT would be performed as stated in the guideline [18]. In spite of the guideline on universal screening, Ruengkhachorn et al. found that compliance to screening was more likely amongst women with two or more risk factors for GDM compared to those with only one risk factor [20].

These studies indicate that even in developed countries adherence to prevailing guidelines for GDM screening and compliance to screening tests seems sub-optimal at best and arbitrary at worst. Very little information, if any, is available on factors that favour or deter screening and provide pointers to what can be done to improve it.

### Barriers for GDM screening

Nielsen et al. interviewed the implementing partners of 11 GDM projects funded by the World Diabetes Foundation in various developing countries and found various challenges in GDM screening and diagnosis including difficulties in screening women during the recommended time period, applicability and relevance of the risk factors used in selective screening programmes, challenges in testing women in the fasting state and need for repeated testing, screening procedure being too time consuming, scarcity of test consumables and lack of equipment etc. [15].

### Determinants of and barriers for GDM treatment

While there are only a few studies looking at determinants and barriers for screening as indicated in the foregoing, studies to assess determinants and barriers for GDM treatment are more extensive. Again, the majority of studies are from high-income countries (13 out of 15).

In their study from Indiana, US, published back in 1992, Marrero et al. examined the specialisation of the health care provider in relation to insulin prescription for GDM and found that family physicians were more likely to initiate insulin therapy than obstetricians [17]. Landon et al. found that the method for glucose surveillance differed greatly among members of the American College of Obstetrics and Gynecologists (ACOG) depending on the post training duration of practice (≥ 15 years or ≤ 15 years) and with members of the Society of Perinatal Obstetricians (SPO). Use of insulin was more frequent among SPO members who also used lower glucose thresholds for initiating insulin compared to ACOG members [16]. Ruggiero et al. investigated the role of social support and dietary and insulin therapy compliance, and found a significant positive correlation; social support accounted for 10 and 24% variance in reported compliance to dietary advice and insulin therapy, respectively [28].

Other studies have taken a more anthropological approach to understanding the determinants and barriers to GDM treatment. Six qualitative studies with focus on women’s experiences with GDM diagnosis were identified in our search. Most women expressed commitment and motivation to manage their diabetes during pregnancy to protect the health of the unborn baby, but behaviour changes, and compliance to treatment were fraught with challenges [29-34]. Two studies found that women interviewed later in their pregnancy had acquired familiarity with their diabetes, and reported greater ability to cope with the stress as the treatment became part of a normal daily routine [31,34]. Persson et al. also noted that such adaptation was easier to achieve for women with GDM in a previous pregnancy [34]. In addition, both Evans & O’Brien and Persson et al. reported that women described their experience as if being controlled by the disease, their families and health care providers; with their behaviour under surveillance, and as if their blood glucose levels determined if they were ‘good mothers’ [31,34] and that delivery was the ‘moment of truth’ when maternal and fetal outcomes were revealed [34]. Lawson & Rajaram reported feelings amongst women with GDM: numb with fear for the baby’s wellbeing, guilt and being personally responsible for the disorder, anxious about the future and distressed with the treatment, losing autonomy when hospitalised and an inability to control the illness. It was also reported that these feelings affected their own perception of self-worth [32]. In a quantitative study from 1996, Persily reported that women who perceived GDM to have a greater stress on their lives were less likely to self-monitor their blood glucose [35]. Hjelm et al. studied perceptions about treatment amongst women with GDM attending a specialised diabetes clinic in Sweden [36]. Limited access and waiting time to meet health care providers coupled with their perceived lack of competence caused anxiety and loss of confidence; whereas provision of adequate information reduced anxiety and gave women a feeling of control over the situation [36]. In another study by Hjelm et al., cost of healthy food and lack of advice and information about exercise in relation to GDM and waiting time to access health care providers for advice were mentioned by women with GDM as important barriers [37]. In her study about food perceptions and concerns among aboriginal women with GDM in Canada, Neufeld points out a number of emotional and psychological challenges related to dietary management during pregnancy: women found it difficult to adhere to the dietary management plan due to pregnancy-related food preferences and cravings, plans being perceived as stressful, adaptation of unhealthy eating patterns such as ‘bulimia and binging’, and frequent use of ‘comfort foods’. Women mentioned that dietary messages were sometimes contradictory, lacked adequate practical information and they did not trust the nutrition education messages received from health care providers [33]. Confusion over dietary recommendations, lack of sufficient advice and concerns about the effects of the recommendations were also pointed out by Hirst et al. in their study from Vietnam [38] as well as the study by Lawson & Rajaram from the US [32]. From a study on immigrant South Asian women with GDM in Australia, Bandyopadhyay et al. reported difficulties in understanding dietary recommendations [29]. In addition, food preferences and cravings, concerns for baby’s growth and fear that too much physical activity would put a strain on the baby were other concerns that hindered compliance to diet and exercise recommendations during GDM pregnancy [29].

Two recent qualitative studies from the US have specifically looked at barriers to treatment [39,40]. Collier et al. conducted focus group discussions with women who had GDM or pre-gestational diabetes during a recent pregnancy. Women mostly reported financial barriers e.g. cost of health care, medical supplies and food, but also barriers related to access to care and insurance, and related to physical activity and maintaining healthy diet; difficulties in finding information and communicating with health care providers; lack of social support; and barriers related to care, e.g. reluctance to inject insulin and treatment being seen as time consuming [39]. Mersereau et al. conducted focus group discussions with health care providers to investigate barriers to good glycaemic control in women with GDM [40]. The barriers identified by the health care providers were lack of knowledge and awareness among the women with GDM, lack of access, including financial and insurance issues, attitudinal barriers such as denial of the severity of the disease, no motivation, lack of compliance, and social barriers. The authors note that most of the health care providers perceived the barriers to be outside their locus of control [40]. Other barriers or challenges noted in studies include difficulty in adhering to a diet when participating in social gatherings [31,34], and insufficient social support hampering compliance to treatment [34]. Finally, Doran & Davis in their study from Tonga highlight structural changes such as more cars leading to reduced physical activity, more unhealthy take away food options and lower rates of home cooking and eating of traditional foods, as factors contributing to unhealthy lifestyle and difficulties in adherence to treatment [30].

Hence, with regard to treatment and factors influencing it, the published literature does provide some direction. The majority of studies focusing on treatment has been qualitative and found that women express motivation and show commitment to manage their diabetes, but that several challenges exist. Social support has been identified in qualitative studies to be key to proper management of diabetes in pregnancy and data from the quantitative study by Ruggiero et al. supports this finding.

### Determinants and barriers for postpartum diabetes screening

The literature dealing with postpartum follow-up of women with GDM is either focussed on postpartum screening for diabetes or prevention of type 2 diabetes in terms of maintaining healthy lifestyles.

### Characteristics of health care providers and health care setting

Screening for diabetes following an index GDM pregnancy is in general reported to be low. Depending on the definition of ‘postpartum diabetes screening’ including the type of test and follow-up period, studies identified in this review – all from the US or Australia – found postpartum diabetes screening rates ranging from 19 to 73% [23,41-45]. Studies that have assessed the frequency of postpartum screening over time show that the proportion of women with GDM completing a postpartum screening test has increased; thus between 1999 and 2004 Dietz et al. found that the proportion of women with confirmed GDM who completed a postpartum screening test within 3 months increased from 9 to 58% in their research setting in north-west US [46]. In their study from Australia, Beischer et al. found that overall postpartum follow-up attendance was 71% and attendance had increased from 43.7% in 1981 to 84.4% in 1995 [47].

A number of studies have examined possible determinants for postpartum screening. Three studies investigated how frequently health care providers screen women with GDM for diabetes postpartum [48-50]. In their study from US, Baker et al. found that only 21% of respondents always screen for diabetes postpartum, whereas Keely et al. reported that 37% of primary care providers in Ottawa, Canada had their GDM patients complete a postpartum OGTT [48,50]. Gabbe et al. reported that 74% of obstetricians and gynaecologists in the US routinely performed a postpartum evaluation of glucose intolerance in women diagnosed with GDM and physicians younger than 40 years of age were more likely to routinely perform postpartum tests (87.6% vs. 73.2%, p = 0.005) [49]. Shah et al. found that internists/endocrinologists in Ontario, Canada ordered the majority of postpartum diabetes screening test and obstetricians the fewest [51], and Stuebe and colleagues found primary care providers being more likely than OBGYN specialists in Massachusetts, US, to order a postpartum screening test for women with a known history of GDM [52]. In contrast, Baker et al. and Kaufmann et al. found in their studies from the US that physicians’ speciality did not significantly influence the likelihood of postpartum testing [48,53].

Morrison et al. assessed two-way interactions and found that being under the care of an endocrinologist insignificantly increased the likelihood of postpartum screening among less educated Australian women, and being seen by a diabetes educator significantly increased the likelihood of postpartum screening among Australian women under the care of an obstetrician [44]. Kim et al. investigated different scenarios to predict postpartum glucose testing in a university hospital in Michigan, USA. After adjusting for confounders the only scenario that significantly predicted testing was a visit to an endocrinologist after delivery [42].

Almario et al. looked at the effect of the gyn/obst-specialists’ practice setting (‘office type’) and found that gyn/obst-specialists working in a high-risk pregnancy setting in the US were more likely to provide postpartum diabetes screening compared to their colleagues working in a normal (non-high risk) pregnancy setting [41]. Likewise, in their study also from the US, Dietz et al. found that practice site was predictive of clinician ordering the test but not for the test being actually taken [46]. Smirnakis et al. studied a population of women with a history of GDM in Massachusetts, US, and found that practice site had a borderline effect on postpartum screening in univariate analysis, but not after adjustment for potential confounders [45]. Moreover, Russell et al. reported that type of referral clinic did not predict postpartum testing in their study from Rhode Island, US [54].

Reminders and advice on postpartum screening have been assessed by four studies. Hence, two studies from Canada found that receiving reminders about testing increased the likelihood of testing [55,56]. Similarly, an Australian study by Morrison et al. found that providing postnatal written information or individualised risk reduction advice significantly increased the likelihood for postpartum screening [44], and in the US, Kim et al. found that women who recalled receiving advice on postpartum screening were more likely to report actually being tested [57].

Finally, the immediate postpartum OGTT was found to be an important predictor of subsequent follow-up test in a study by Beischer et al. from Australia. The study moreover noted that whereas patients attending a public clinic were more likely to receive postpartum OGTT, private patients who received the postpartum test were more likely to be enrolled in the long-term follow-up programme [47].

The studies focusing on identifying health care provider- or health care setting-related determinants for postpartum screening show that often postpartum screening rates are sub-optimal. Studies that focus on the health care provider or setting seem not to provide a clear picture, but this may be due to different practice between countries and/or over time. Yet, receiving reminders or postnatal information appear to have a positive impact on the likelihood of postpartum screening, as does recollection of receiving advice on postpartum testing. For longer-term follow-up it has been indicated that the immediate postpartum test and attendance in a private clinic may be predictors, at least in Australia.

### Barriers according to health care providers

Three studies – two from the US and one from Australia - reported on health care providers’ perceptions of barriers to performing postpartum diabetes screening [48,50,52]. Barriers mentioned were diverse: not seeing the patient, the patient being lost to follow-up and lack of communication/collaboration between health care providers were the most widely mentioned issues. Other barriers mentioned were inconsistent guidelines or lack of familiarity to guidelines; not aware about history of GDM; patients not considering the test necessary, or declining testing, or unable to complete test; testing not affordable; patient uninformed or lack understanding of need for test, and practice being too busy etc. [48,50,52].

### Characteristics of the client with GDM

Studies from high-income countries have also focused on client characteristics and other determinants for postpartum diabetes screening.

Studies from the US conducted by Almario et al. and Ferrara et al. found that women had an increased likelihood of being screened postpartum if their GDM was diagnosed early - before 24 weeks and 20 weeks of gestation, respectively - compared to women later in pregnancy [41,58]. In contrast, Hunt & Conway did not find a significant association between gestational age at diagnosis and glucose testing postpartum [59]; but they found that women missing the postpartum testing had higher fasting and 1-h OGTT glucose levels at GDM diagnosis [59]. Almario et al. noted that women diagnosed with a GCT had an increased likelihood of postpartum screening compared to women diagnosed with GDM using a 3-h 100 g OGTT [41]. Blatt et al. found that women who did not return for postpartum testing had significantly lower glucose concentration in their 2-h OGTT than those who returned [23]. Other studies from the US found no significant effect of the results of the fasting or the OGTT on postpartum screening irrespective of the glucose load [42,58]. Smirnakis et al. in their study from Massachusetts, US [45] also found no significant effect of the 1-h, 2-h and 3-h OGTT results, but found that women with glucose concentrations ≥ 98 mg/dl (5.4 mmol/l) in their fasting glucose test or ≥171 mg/dl (9.5 mmol/l) at 1-h at the 50 g GCT were more likely to undergo postpartum screening than were women with concentrations below these levels. Similarly Beischer et al. from Australia found that the results of the 50 g GCT significantly predicted both enrolment and maintenance in postpartum follow-up screening program [47]. Kwong et al. however found no significant association between the results of the 50 g GCT and postpartum screening in their study from Canada [60]. Studying a population in the US, Hunt & Conway found that women not returning for postpartum testing had higher mean fasting, preprandial and 2-h postprandial glucose levels during pregnancy [59]. Kim et al. included total number of prenatal visits in their study model conducted on data from Michigan, but found that it did not predict postpartum glucose testing when included in multivariate analysis [42]. Dietz et al. and Hunt & Conway have both looked at the timing of the test during pregnancy, but found that it neither influenced postpartum test order and test completion [46] nor whether the women returned for postpartum screening [59]. On the other hand attending a postpartum visit with an OBGYN provider was found to greatly increase the likelihood of postpartum glucose testing in two other studies from the US [43,54] and if conducted within 3 months of delivery it also increased the likelihood of completing the test [46]. Multiple visits were likewise shown to be positively associated with postpartum testing [42,58].

Ten studies have assessed the effect of treatment type for GDM on postpartum glucose testing. Six of these were from the US, including the study by Almario et al. which found that women receiving pharmacotherapy during pregnancy (treated with insulin or glyburide as a combined variable) were more likely to be screened compared to women treated with diet [41]. Hunt & Conway on the other hand found that women who failed to return for postpartum testing were more likely to have had required medication to treat their GDM [59]. Dietz et al. investigated predictors for physician ordering postpartum glucose testing and for test completion and found that use of insulin or glyburide during pregnancy was not significantly associated with these outcomes [46]. Studies from the US have also looked at the use of insulin and glyburide as two independent variables. Ferrara et al. and Hunt & Conway looked at glyburide, but whereas Ferrara et al. found that the use of glyburide increased the likelihood of postpartum glucose testing [58] Hunt & Conway did not find a statistically significant difference in the use of glyburide among women who returned and did not return for postpartum testing [59]. Six studies have looked at insulin use, but their results differ: two studies from the US and one from Australia found no significant association with use of insulin and postpartum glucose screening [42-44], one study from the US and two studies from Canada found on the other hand that insulin significantly predicted postpartum glucose testing [56,58,60]. In addition, Beischer et al. found that requiring insulin during pregnancy predicted enrolment and maintenance in follow-up program for postpartum testing in Australia [47], but Hunt & Conway found that women in their US population who failed to return for postpartum testing were more likely to have used insulin [59]. Finally, Lawrence et al. found that the use of oral agents alone decreased the likelihood of testing among their study population from Southern California [43].

Studies from the US looking at self-monitoring [59] and nutrition visit during pregnancy [46], as well as a Canadian study focusing on HbA1c level during pregnancy [60] found no significant association with postpartum testing.

When assessing obstetrical history as a predictor for postpartum testing studies found no significant associations with gravidity [42,53], number of prior pregnancies with GDM [41], prior macrosomia [59], multiple births with affected pregnancy [42], prior history of preeclampsia or eclampsia [42]. When it comes to the role of parity [43,46,53,54,58-60], study results from the US and Canada, are inconsistent. Kaufmann et al. included subsequent pregnancies in their analysis on US data but found no association with postpartum testing [53]. Hunt & Conway found that women who failed to return for postpartum glucose testing were more likely to have a history of GDM compared to women who returned [59], but other studies from the US as well as from Australia and Canada investigating the effect of previous GDM on postpartum testing did not find statistically significant associations [42,44,54,56,60].

Studies from the US have also investigated whether occurrences in the current GDM pregnancy influences the likelihood of postpartum glucose testing, but the findings are no different from studies looking at occurrences in past pregnancies, and no significant association is found between preeclampsia [59], gestational week at delivery [42,54,59], having a caesarean delivery [43,54,59], macrosomia [42,46,58,59], live birth [42], need for neonatal intensive care [54] and postpartum diabetes testing. However, Lawrence et al. found that year of delivery significantly influenced the likelihood of postpartum glucose testing within 6 months from delivery, with women delivering in the years 2000 to 2006 having a higher likelihood for testing than women in 1999 [43]. The Australian study by Beischer et al. similarly found that the year of the pregnancy influenced the likelihood of enrolment in postpartum testing programme [47].

Two studies from the US investigated the effect of weight gain during pregnancy and one looked at weight changes in general, but none of them found a significant association with postpartum testing [42,53,59]. Hunt & Conway in addition found that women who did not return for postpartum glucose testing weighed more prior to pregnancy than those who returned [59]. Other studies focusing on maternal weight likewise found that obesity or increasing weight significantly reduced the likelihood of testing [23,58]. Studies focusing on BMI are available from US, Australia and Canada; however, they have in general not found any significant association with postpartum glucose testing. Hence, only one – a study from Canada - of the nine studies assessing BMI as a predictor for screening [41,42,44-46,54,56,59,60] found a significant association with women being normal or overweight being more likely to go for any postpartum glucose testing than obese women [56].

Equally variable results are seen when it comes to assessing whether age, income, education, ethnicity/race and marital status are predictors of screening [23,41-47,53,54,56,58-60]. One study from Australia and two from the US investigated effect of primary language and found no significant association [44-46]. Two studies looked at country at birth as a predictor for postpartum testing and whereas Morrison et al. in their study from Australia found no effect of whether one was born in or outside Australia [44], Lawrence et al. in their study from the US found that women born outside the US had significantly higher odds for testing compared to US born women [43]. Family history of type 2 diabetes did not predict postpartum glucose testing according to five of the six studies who included it in their analysis [41,42,44,56,59,60]. Studies have also looked at employment status [44], antenatal tobacco use [54], changes in diet [53], changes in exercise [53], and health insurance status [45,54] without finding significant associations with postpartum screening.

No studies from low- or middle-income countries have examined determinants for postpartum screening, but studies from high-income countries investigating this have shown inconsistent results or only found statistically insignificant associations when it comes to determinants related to the GDM screening test conducted during pregnancy, treatment, obstetric history, current pregnancy, demography, weight, lifestyle changes, family history of type 2 diabetes, and socio-economic factors. However, having a postpartum visit with an OBGYN provider seem to increase the likelihood of postpartum testing and the likelihood is further increased with more postpartum visits.

### Barriers according to women with GDM

Only two studies - one survey and one qualitative study - asked women with a history of GDM about barriers to postpartum diabetes screening [50,61]. Keely et al. investigated why women in Ottawa, Canada with a history of GDM did not complete postpartum screening for diabetes using an OGTT. By far the most frequent reason was time pressure, but lost requisition was mentioned by almost 20% respondents [50]. In the qualitative study by Bennet et al. conducted among women with GDM attending a high risk obstetric clinical practice in Baltimore, US, a number of themes were identified including recent delivery experience and baby’s health issues; adjustment to the new baby (emotional stress, feeling overwhelmed and lack of time and burden of child care); concerns about postpartum and future health (feeling healthy and not in need for care, and fear of receiving bad news); and experiences with medical care and services (dissatisfaction with care and logistics of accessing care) as barriers to postpartum follow-up care [61].

### Determinants and barriers for healthy postpartum lifestyle and prevention of future diabetes

Studies have also investigated determinants for maintaining a healthy lifestyle (diet or exercise) after an index GDM pregnancy. Hoedjes et al. reported in their study from the Netherlands that although women expressed that they intended to live a healthy postpartum lifestyle, it was generally not achieved [62]. This notion is supported by two studies from Australia which found that among women with GDM in the past 6–24 months unhealthy diet was prevalent [63] and only 33.6% reported sufficient physical activity [64]. A reason may be that women with a history of GDM do not perceive themselves to be at increased risk of future diabetes. Hence, Kim et al. examined risk perception for diabetes among women with a history of GDM in a US population and found that although 90% of women recognized GDM as a risk factor for future diabetes, only 16% believed they themselves were at high risk of developing diabetes, though the proportion increased to 39% when asked to estimate their risk assuming they maintained their current lifestyle [65]. In a study from Denmark, Stage et al. found that 40% of women with a history of GDM were very worried about developing diabetes in the future, 46% were a little worried and 14% were not worried at all [66]. In addition, whereas Stage et al. found no correlation between worrying and postpartum weight loss, Kim et al. found that women who perceived themselves to be at no or slight risk of diabetes were less likely to modify their lifestyle [65,66]. In Neufeld’s study among aboriginal women with a history of GDM or currently experiencing a GDM affected pregnancy in Canada it was noted that many women tried to continue eating healthy postpartum to protect their health; however, some postpartum women felt they no longer had to worry about what they were eating as their dietary intake would no longer impact the health of the baby [33].

The importance of self-efficacy and social support for postpartum healthy lifestyle has been investigated for both diet [63,67] and physical activity [64,67,68]. Zehle and colleagues investigated cognitive and social factors, including the role of self-efficacy and social support, related to postpartum dietary behaviours among women with recent GDM in Australia [63]. They found that non-English speaking women consumed less vegetables and bread and more fried food than English speaking women. Self-efficacy was associated with high vegetable consumption, ability to cook healthy foods, and reporting that healthy diet is not a difficult change and that dislike of healthy foods by other household members is not a barrier for them. Moreover, self-efficacy when busy and not reporting a dislike of healthy foods by others at home were associated with high fruit consumption [63]. Focusing on physical activity, Smith and colleagues found that in multivariate analysis sufficient physical activity was independently associated with high self-efficacy and social support; yet more than half of the women in their Australian study reported never receiving support in the form of assistance with household work or others exercising with them [64]. Another Australian study by Koh et al. found a similar significant association of social support and self-efficacy for physical activity, but noted that these concepts could only explain a small proportion of the variance in physical activity among women with recent GDM in their study population [68]. In their study from the US, Kim and colleagues examined the association between self-efficacy and social support for both physical activity and diet and found similar results except that the adjusted association between self-efficacy and dietary quality just missed significance [67]. The role of various socio-demographic variables in physical activity and weight management among women with recent GDM has been investigated by two Australian studies but no significant association was noted [68,69].

When it comes to continuing lifestyle modifications postpartum, studies indicate that intention may be there, but many women do not succeed in continuing their modifications. This may be influenced by their perception of risk of future diabetes and particularly by self-efficacy and social support.

### Barriers for healthy postpartum lifestyle

A number of studies from Australia, US and the Netherlands have investigated barriers to a healthy postpartum lifestyle among women with history of GDM [62,64,70-73]. Lack of time and/or energy was a common barrier mentioned in all studies [29,62,64,70-73] and so was lack of child care support [62,70-73]. Other barriers identified in the studies included not feeling well and/or emotional distress [72,73]: lack of motivation [70,71]; financial barriers [70,71]; other domestic responsibilities such as cooking [72]; lack of knowledge [62,64]: lack of understanding about GDM [62]; lack of social support [64,70-72] lack of health care provider support [62]; feeling of solitude, dullness and isolation from family and friends [62,70]; poor body image [70]; bad weather [70]; considering oneself to be too young to be on a restricted diet [29]; obstacles at work [71]; unsuitable local neighbourhood or no access to exercise equipment [64,71]; cultural expectations e.g. needs of women come last in the family [70,72]; and lack of enjoyment of physical activity [64]. Moreover, in a study among immigrant women in Australia it was mentioned that breastfeeding was a barrier to postpartum weight reduction as it made them increase their food intake [29].

## Discussion

This review of scientific literature on barriers and determinants for screening, diagnosis, management and post-partum follow up of GDM pregnancies examined 58 full text papers published in peer reviewed journals. In the final stage of literature review we excluded 3 non-English articles. This of course may entail some bias, missing out some of the additional local issues that may have been highlighted in these articles. The decision to limit the search to not include grey literature, reports and book chapters was made due to allowed journal space for a review article.

The review does not cover determinants and barriers to ANC attendance in general. GDM screening will only occur when women are able to overcome barriers to ANC attendance in the first place. Others have published extensively on determinants and barriers for ANC attendance [74,75].

There are serious barriers to proper GDM services, management and care even in high-income countries and across study settings (see Table 4). Although some determinants and barriers to GDM management are consistent across studies, many are contextual or culture-specific. The barriers operate at different levels – cultural and societal; health system resources; health care provider and client characteristics. Lack of knowledge and perceived seriousness about the issue amongst policy makers, health care providers, affected women and their family and lay people in general is perhaps the biggest hurdle. Compartmentalisation of care is another important barrier - as noted earlier- not seeing the patient, the patient being lost to follow-up and lack of communication/collaboration between health care providers were the most widely mentioned issues by the health care providers as barriers to postpartum follow up. Following delivery women with GDM usually no longer have diabetes and are no longer pregnant and therefore unlikely to visit physicians or gynaecologists for check-ups and thus perceived to be lost to follow-up. However these women do visit health services focused on the wellbeing of their babies, for instance for the child’s vaccination program and follow-up and are likely to do so at regular intervals for at least five years. So why cannot this opportunity be used to provide them follow-up advice and conduct necessary tests? Why cannot the health system tag the mother’s GDM status to the child for the benefit of both? [76]. Studies have shown that lifestyle modifications and/or pharmaceutical intervention following a GDM pregnancy is as effective in preventing or delaying the onset of type 2 diabetes as in the case of other people with pre-diabetes [12,13,77-79]. Clinical trials now provide grade A evidence for the impact of multiple interventions to prevent the progression to type 2 diabetes in women with a history of GDM. Both lifestyle modification and pharmacological therapies (metformin, troglitazone, and pioglitazone) have been shown to reduce diabetes development by 50% or more [80]. Several prospective studies have been initiated in different ethnic groups to replicate the findings of these studies with encouraging initial positive results [81-83]. The diagnosis of GDM should initiate a long-term intervention and diagnostic process to minimize the risk of developing diabetes or to diagnose it as early in the course of disease as possible [80]. Moreover, women with one GDM affected pregnancy are at high risk of developing GDM in subsequent pregnancies [84]. The need to address barriers to postpartum follow-up and lifestyle modifications are therefore imperative. In view of the emerging burden of GDM in populous low- and middle-income countries, it is remarkable that all except four of the included studies were conducted in high-income countries. It is equally remarkable that few studies sought to identify barriers and how they might be overcome by including the pregnant and postpartum women’s opinions and propositions, including the role of breastfeeding.

**Table 4:** Barriers identified in high-income and low- and middle-income countries

**High-income countries**	**Low- and middle-income countries**
• No studies focusing on barriers to GDM screening and diagnosis were identified.	• Barriers to GDM screening and diagnosis include difficulties in screening women during the recommended time period, applicability and relevance of the risk factors used in selective screening programmes, challenges in testing women in the fasting state and need for repeat test, screening procedure being too time consuming, scarcity of test consumables and lack of equipment.
• Barriers to treatment include lack of social support, stress, cost of healthy food, cost of health care and medical supplies, lack of advice and information about diet and exercise, dietary messages being contradictory, dietary messages being difficult to understand, lack of trust in messages received from the health care providers, waiting time to access health care providers for advice, lack of access to health care and health insurance, pregnancy-related food preferences and cravings, diet plans being perceived as stressful, adaptation of unhealthy eating patterns such as bulimia and binging, frequent use of ‘comfort foods’, difficulty in adhering to a diet when participating in social gatherings, concerns for baby’s growth and putting a strain on the baby, reluctance to inject insulin, treatment being time consuming, lack of knowledge, denial of severity, lack of motivation, other social barriers.	• Barriers to treatment include confusion over dietary recommendations, lack of sufficient advice, concerns about the effects of the recommendations, structural changes such as more cars leading to reduced physical activity, more unhealthy take away food options and lower rates of home cooking and eating of traditional foods.
• Barriers to postpartum screening include health care provider not seeing the patient, the patient being lost to follow-up, lack of communication/collaboration between health care providers, inconsistent guidelines or lack of familiarity to guidelines, not aware about history of GDM, patients not considering the test necessary, or declining testing, or unable to complete test, testing not affordable, patient uninformed or lack understanding of need for test, practice being too busy, time pressure (women), lost requisition, recent delivery experience, baby’s health issues, adjustment to the new baby (emotional stress, feeling overwhelmed and lack of time and burden of child care), concerns about postpartum and future health (feeling healthy and not in need for care, and fear of receiving bad news), and experiences with medical care and services (dissatisfaction with care and logistics of accessing care).	• No studies focusing on barriers to postpartum GDM screening were identified
• Barriers to healthy postpartum lifestyle include lack of time and/or energy, lack of child care support, not feeling well, emotional distress, lack of motivation, financial barriers, domestic responsibilities such as cooking, lack of knowledge, lack of understanding about GDM, lack of social support, lack of support from health care provider, feeling of solitude, dullness and isolation from family and friends, poor body image, bad weather, considering oneself to be too young to be on a restricted diet, obstacles at work, unsuitable local neighbourhood, no access to exercise equipment, cultural expectations, lack of enjoyment of physical activity, breastfeeding as it made some women increase their food intake.	No studies focusing on barriers to healthy postpartum lifestyle were identified

Studies on the magnitude of the GDM burden in recent years have shown that GDM is a fast growing problem in low- and middle-income countries. Studies from South India and China found prevalence rates of 17.8% and 17.5%, respectively [85,86]. At the same time many developing countries are experiencing rapidly increasing diabetes prevalence rates [87] and continue to struggle with high rates of maternal mortality and morbidity and inadequate emergency obstetric care [88]. Availability, affordability and access to services for GDM are likely to be even bigger barriers in low- and middle-income countries than what has been illustrated in this review. GDM increases the risk of macrosomia, shoulder dystocia and obstructed labour and so pregnancies complicated by GDM without proper access to obstetrical care can be fatal or result in debilitating outcomes. Fortunately, a number of developing countries have started to pilot or implement GDM programmes [15]. Health care providers, health care planners, public health professionals and policy makers need to understand and take these barriers into consideration to ensure proper initiatives to address GDM are put in place, without forgetting to involve the women at risk.

## Conclusions

This systematic review has evaluated determinants and barriers to proper GDM services. Very few studies from low- and middle-income countries were found. Compliance to screening tests was sub-optimal, but little information is available on what factors influence poor compliance and thereby identify what can be done to improve it. While women express commitment and motivation for treatment to protect their health and thereby the health of the unborn baby, behaviour changes and compliance to treatment are associated with challenges and a number of barriers have been identified, particularly in the qualitative studies. Also postpartum screening for diabetes is unsatisfactory and a number of determinants and barriers have been identified, including patients being lost to follow-up, lack of time and lost requisition etc. Variable results were found for many determinants. Following a recent GDM pregnancy many women desire and intend to maintain healthy lifestyles to prevent future diabetes but find the effort challenging. Self-efficacy and social support are important determinants in this regard. Understanding determinants and barriers within the local context is vital in designing public health interventions to address the growing burden of GDM and diabetes. Therefore, studies from low- and middle-income countries where the prevalence of GDM is rapidly increasing are especially warranted.

## Competing interests

The authors have no competing interests to declare.

## Authors’ contributions

KKN, AK, PD, MdC, ICB designed the study; KKN carried out the literature search and selection; KKN, AK, PD, MdC, ICB contributed to the analysis; KKN wrote the first draft of the manuscript; KKN, AK, PD, MdC, ICB critically revised the manuscript and approved the final version.

## Pre-publication history

The pre-publication history for this paper can be accessed here:

http://www.biomedcentral.com/1471-2393/14/41/prepub
